# Toni Mochty: Bardet Biedl syndrome “avant la lettre”

**DOI:** 10.1111/cge.13647

**Published:** 2020-02-16

**Authors:** Wouter de Herder

**Affiliations:** ^1^ Department of Endocrine Oncology Erasmus MC Rotterdam The Netherlands

The names of the French physician Georges Louis Bardet (1885‐1966) and the Hungarian pathologist‐endocrinologist Artur Biedl (1869‐1933) are associated with the Bardet‐Biedl syndrome (BBS) on which Bardet published in 1920^1^ and Biedl in 1922.^2^ This autosomal recessive syndrome comprises truncal obesity, cognitive impairment, polydactyly, rod‐cone dystrophy (RCD), hypogonadotropic hypogonadism and renal abnormalities. Secondary features of BBS include: speech delay—disorder, developmental delay, ataxia—poor coordination—imbalance, behavioral abnormalities, brachydactyly—syndactyly, mild hypertonia, craniofacial dysmorphism, oro‐dental abnormalities, cardiovascular anomalies, hepatic involvement, Hirschsprung disease, female genitourinary malformations, anosmia and diabetes mellitus. At least 23 genes are associated with BBS.^3^

Anton (Toni) Mochty was born in the small farmers community of Haindorf, Austria in 1886 and from the age of 10 he was “exhibited” in Europe as “the fattest boy in the world” and “a wonder of human nature,”^4^ (Figure 1). His manager was the former sorcerer Heinrich Tischer (1868‐1928). According to the newspapers, his mother nursed him until his fifth year because “he took little solid food” before and at the same age he began to creep. He was “by no means a bright boy.” At the age of 10, he was 1.50 m. (5 ft.) tall and weighed 150 kg. He had six toes on each foot and six fingers on each hand. In his village, they called him the “rubber ball,” on account of the “rotundity of his features.” He had numerous brothers and sisters who all had a normal number of toes and fingers and no developmental disorders.^4^ In July 1912, Tischer assisted Toni (aged 25, or 26) to board a train, but both fell. The manager got seriously injured and Toni disappeared from the scene (as reported by the forensic pathologist Richard Kockel—1865‐1934). Already in 1896, the title of “fattest boy in the world” was overtaken by a 9‐year‐old Charley Bilcher from the United States.

**Figure 1:**
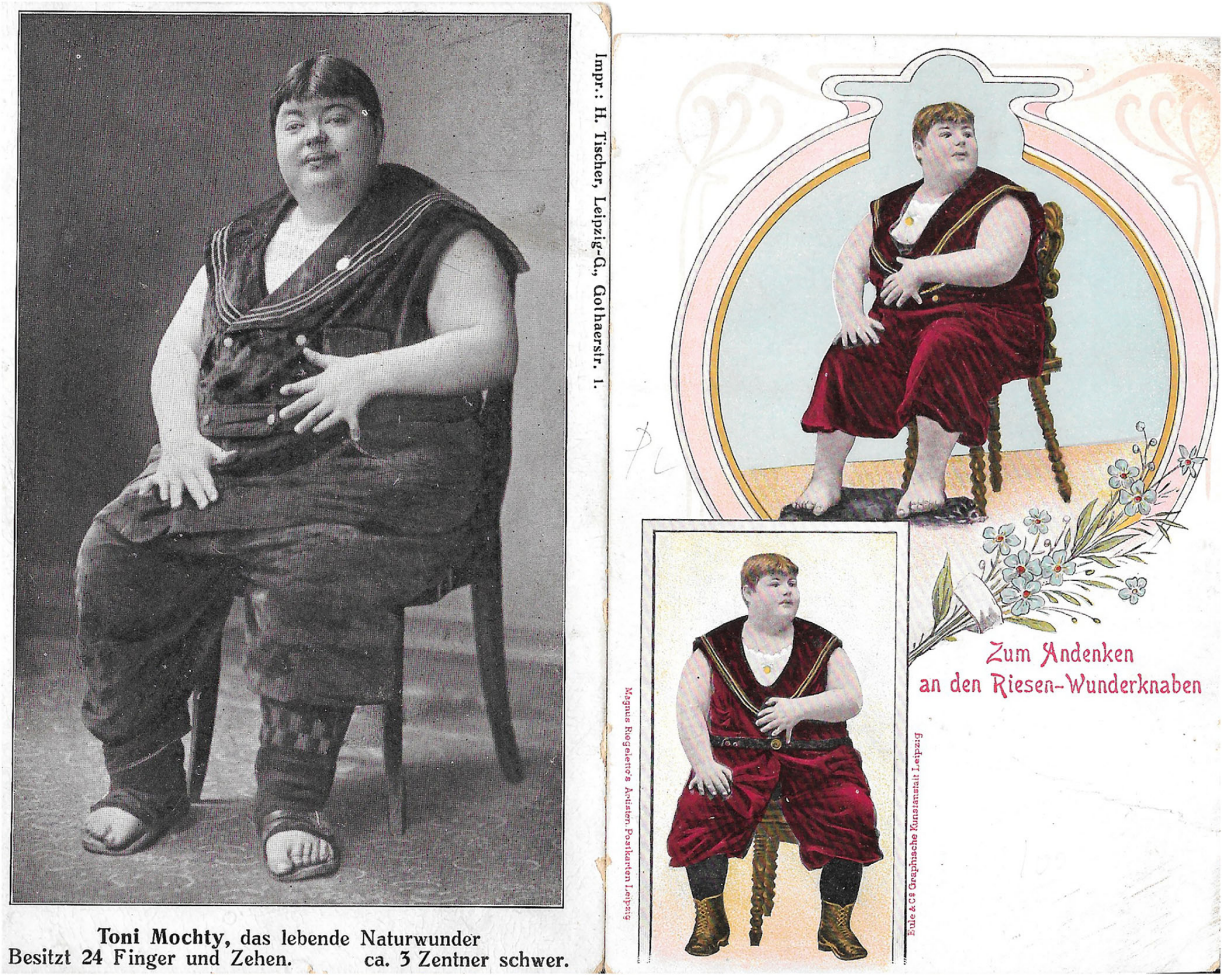
Toni Mochty, Picture Postcard (L) and Advertising Postcard (R). Collection W.W. de Herder [Colour figure can be viewed at http://wileyonlinelibrary.com]

Anton/Toni Mochty had several clinical features which are in line with the diagnosis of an autosomal recessive ciliopathy and most probable BBS: bradyphrenia—slow development, truncal obesity, achalasia and polydactyly. However, information on disturbed visual acuity in line with RCD in this case is regretfully lacking. Bardet and Biedl apparently were not aware of his existence and as such, he presented with BBS “avant la lettre.”

## Data Availability

REF 1. The data that support the findings of this study are openly available at: https://fr.wikipedia.org/wiki/Georges_Bardet. REF 2. The data that support the findings of this study are openly available in Zur Geschichte der Endokrinologie und Reproduktionsmedizin pp 50‐51 at: https://link.springer.com/chapter/10.1007/978-3-642-79152-9_20. REF 3. The data that support the findings of this study are openly available in Eur J Hum Genet. at: https://www.ncbi.nlm.nih.gov/pmc/articles/PMC3522196/pdf/ejhg2012115a.pdf. REF 4. The data that support the findings of this study are openly available at: https://newspapers.library.in.gov/cgi-bin/indiana?a=d&d=GER18960116; https://www.newspapers.com/newspage/338630407/; https://www.newspapers.com/newspage/53951906/.
